# Potato NAC Transcription Factor StNAC053 Enhances Salt and Drought Tolerance in Transgenic *Arabidopsis*

**DOI:** 10.3390/ijms22052568

**Published:** 2021-03-04

**Authors:** Qi Wang, Cun Guo, Zhiyuan Li, Jinhao Sun, Zhichao Deng, Lichao Wen, Xiaoxu Li, Yongfeng Guo

**Affiliations:** 1 Key Laboratory for Tobacco Gene Resources, Tobacco Research Institute, Chinese Academy of Agricultural Sciences, Qingdao 266101, China; 82101181074@caas.cn (Q.W.); 82101172197@caas.cn (C.G.); 82101181071@caas.cn (Z.L.); 82101181072@caas.cn (J.S.); 82101192207@caas.cn (Z.D.); 82101182201@caas.cn (L.W.); 2 Graduate School of Chinese Academy of Agricultural Sciences, Beijing 100081, China

**Keywords:** potato, NAC transcription factor, ABA, abiotic stress

## Abstract

The NAC (NAM, ATAF1/2, and CUC2) transcription factors comprise one of the largest transcription factor families in plants and play important roles in stress responses. However, little is known about the functions of potato NAC family members. Here we report the cloning of a potato NAC transcription factor gene *StNAC053*, which was significantly upregulated after salt, drought, and abscisic acid treatments. Furthermore, the StNAC053-GFP fusion protein was found to be located in the nucleus and had a C-terminal transactivation domain, implying that StNAC053 may function as a transcriptional activator in potato. Notably, *Arabidopsis* plants overexpressing *StNAC053* displayed lower seed germination rates compared to wild-type under exogenous ABA treatment. In addition, the *StNAC053* overexpression *Arabidopsis* lines displayed significantly increased tolerance to salt and drought stress treatments. Moreover, the *StNAC053-OE* lines were found to have higher activities of superoxide dismutase (SOD), catalase (CAT), and peroxidase (POD) under multiple stress treatments. Interestingly, the expression levels of several stress-related genes including *COR15A,* *DREB1A*, *ERD11*, *RAB18*, *ERF5*, and *KAT2*, were significantly upregulated in these *StNAC053*-overexpressing lines. Taken together, overexpression of the stress-inducible *StNAC053* gene could enhance the tolerances to both salt and drought stress treatments in *Arabidopsis*, likely by upregulating stress-related genes.

## 1. Introduction

Abiotic stresses such as high salinity, drought, and extreme temperatures negatively affect plant growth and development, causing yield loss in crop plants [1]. In order to survive adverse environmental conditions, plants have developed various adaptive mechanisms that involve changes at both physiological and biochemical levels during evolution [2]. The molecular mechanisms that cope with abiotic stresses have been studied extensively in plants, and transcriptional regulation of gene expression exerts a significant role in this process. Members of several transcription factor families in plants, including NAC, WRKY, MYB, and bZIP, have been functionally characterized to be involved in transcription regulation of plant stress responses [2,3,4,5].

NAC proteins constitute one of the largest plant-specific transcription factor families, and have been shown to be involved in various developmental processes in plants, including leaf senescence [6,7], secondary wall formation [8,9], lateral root development [10,11,12], shoot apical meristem development [13,14], floral development [15], plant hormone signaling [16,17,18], and cell division [19]. Plant NAC proteins contain a highly conserved DNA-binding domain named NAC domain in the N-terminal region and a variable transcription regulatory domain in the C-terminal region. So far, a large number of NAC transcription factors have been successively identified in different plant species, including 117 genes in *Arabidopsis*, 151 in rice (*Oryza sativa*) [20], 110 in potato (*Solanum tuberosum*) [21], 168 in durum wheat (*Triticum turgidum* L. ssp. Durum) [22], 152 in tobacco (*Nicotiana tabacum*) [23], 152 in soybean (*Glycine max*) [24], and 82 in melon (*Cucumis melo*) [25].

It has been reported that some *NAC* genes exert central roles in plants’ response to various abiotic stresses, including in the model plant *Arabidopsis* and crop plants such as rice (*Oryza sativa*), soybean (*Glycine max*), wheat (*Triticum aestivum*), and maize (*Zea mays*). ANAC019, ANAC055, and ANAC072 were demonstrated to significantly increase drought tolerance in transgenic *Arabidopsis* via binding to and activating the *ERD1* gene, whereas ANAC069 and AtNAP function to negatively regulate salt stress tolerance through reducing ROS scavenging ability and repressing *ARBE1*, respectively [26,27,28]. In rice, the expression of *SNAC3* was significantly induced by drought, salt, high temperature stress and ABA treatment, and its overexpression resulted in increased drought and high-temperature stress tolerance [29]. OsNAC10, an ABA-dependent NAC transcription factor, could enhance tolerance against drought stress and improve grain yield under field drought conditions in transgenic rice [30]. In rice, *ONAC022*-overexpressing transgenic plants showed an increased tolerance against salt and drought stresses via an ABA-mediated pathway [31]. Two soybean NAC proteins, GmNAC11 and GmNAC20, were identified to be able to strongly improve tolerance against high salinity stress in transgenic plants. Meanwhile, *GmNAC20*-overexpression also led to increased tolerance to freezing stress [32]. In wheat, overexpression of *TaNAC2* conferred strong drought, salt and freezing stress tolerance to transgenic plants [33]. The stress-inducible *TaNAC4* was found to function as a transcriptional activator involved in wheat responses to biotic and abiotic stresses [34,35]. Ectopic expression of *TaSNAC11-4B* in *Arabidopsis* promoted ROS accumulation and significantly accelerated drought and ABA-induced leaf senescence [36]. As a positive regulator in maize’s response to drought stress, *ZmNAC111* expression was found to be higher in drought-tolerant lines and lower in maize plants that were more sensitive to water-stressed conditions [37]. When a maize drought-responsive NAC transcription factor, ZmNAC55, was used to transform *Arabidopsis*, the transgenic plants showed significantly enhanced drought tolerance [38]. *ZmSNAC1* is another stress-responsive gene that enhanced drought tolerance in transgenic *Arabidopsis* [39]. Overall, results from these studies showed that NAC proteins play important roles in plants’ response to abiotic stresses, and changes in their expression can alter abiotic stress tolerance in plants.

Potato (*Solanum tuberosum* L.) is one of the most important horticultural crops and also the fourth principal food crop. The yield of potato is often affected by multiple abiotic stresses, especially drought and salt stresses. Higher tolerance to drought and salt stresses is thus among the major targets in potato breeding. In this study, StNAC053, an NAC transcription factor from potato, was cloned and functionally characterized. Its expression pattern analysis indicated that *StNAC053* was induced by high salinity, drought and ABA treatments. StNAC053 protein was found to be localized in the cell nucleus and could function as a transcriptional activator. *Arabidopsis* plants overexpressing *StNAC053* were generated in this study, which showed enhanced tolerances to drought and high-salinity stresses compared to WT. These results suggested that StNAC053 play an important role in potato’s response to drought and high salt stresses.

## 2. Results

### 2.1. Isolation and Characterization of the StNAC053 Gene

**Figure 1:**
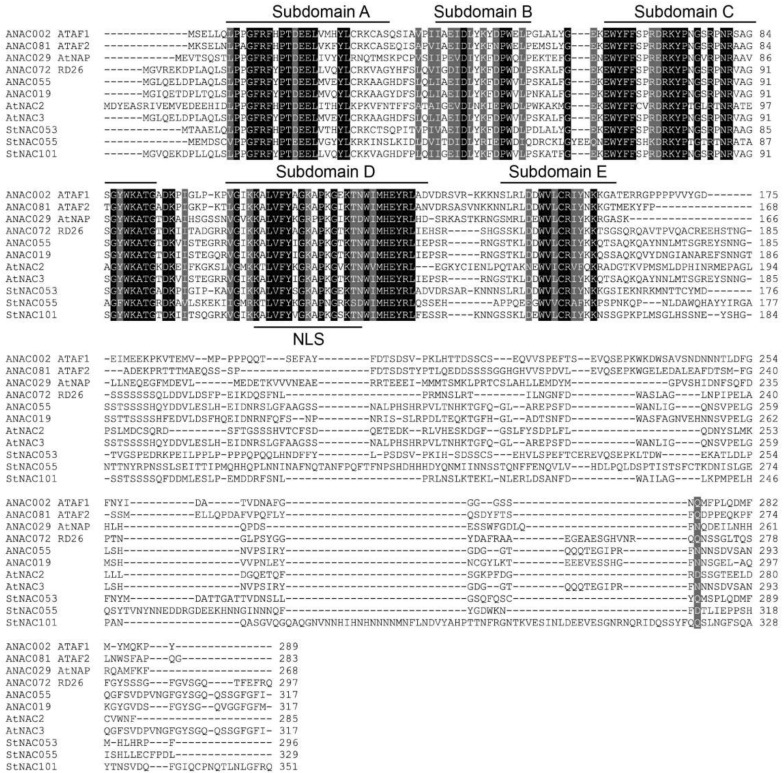
Multiple sequence alignment of StNAC053 with ten other NAC proteins from *Arabidopsis* and potato. The locations of nuclear location signal (NLS) and the five subdomains (A–E) are indicated by black lines.

In the current study, an *NAC* gene was cloned from potato. According to its chromosomal localization, it has been designated as *StNAC053* (GenBank: EU049847) in a previous study [21]. The full length CDS of *StNAC053* is 891 bp that encodes a 296 amino acid protein. The molecular weight (MW) and theoretical isoelectric point (pI) of the protein are 33,898.5 Da and 6.929, respectively. Multiple sequence alignments showed that StNAC053 possesses a highly conserved NAC domain (8-132) at the N-terminus and a transcription activation region at the C-terminus. The NAC structure contained five subdomains (A, B, C, D, E) which have been predicted to function as a DNA-binding domain. Further, a nuclear localization signal (NLS) was identified in the subdomain D (Figure 1).

### 2.2. Phylogenetic and Motif Analysis of the StNAC053 Protein

**Figure 2:**
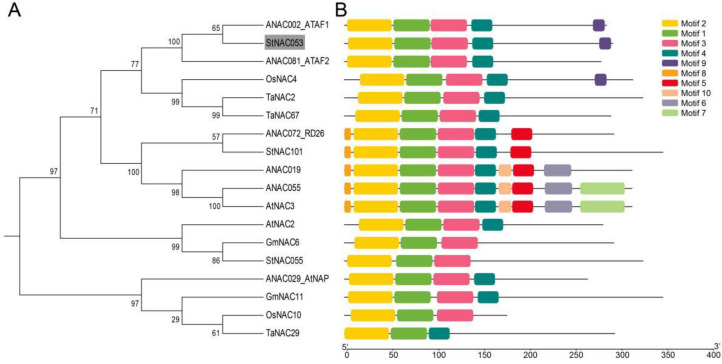
Phylogenetic analysis and motif organizations of StNAC053 and homologous NAC proteins. (**A**) The phylogenetic tree was generated from the alignment of StNAC053 and other plant NAC proteins using the neighbor-joining (NJ) method. (**B**) The motif organizations were predicted using MEME.

To investigate the evolutionary relationship between StNAC053 and other NAC proteins, we constructed a neighbor-joining tree of the StNAC053 protein sequence with 17 other plant NAC proteins from *Arabidopsis*, wheat, rice, soybean, and potato. As a result, StNAC053 was clustered together with ATAF1/ANAC002 and ATAF2/ANAC081 (Figure 2A), which are both abiotic stress-responsive genes [40]. Subsequently, the MEME online tool was used to analyze the conserved domains of StNAC053 and other plant NAC proteins. A total of 10 conserved functional domains were identified (Supplementary Figure S1). Among them, motif 2 represents the subdomain A and subdomain B. Motif 1 corresponds to subdomain C. Motif 3 corresponds to subdomain D. Motif 4 corresponds to subdomain E (Figure 2B). These results indicated that StNAC053 encodes a stress-responsive NAC transcription factor.

### 2.3. Expression Patterns and Promoter Analysis of StNAC053

**Figure 3:**
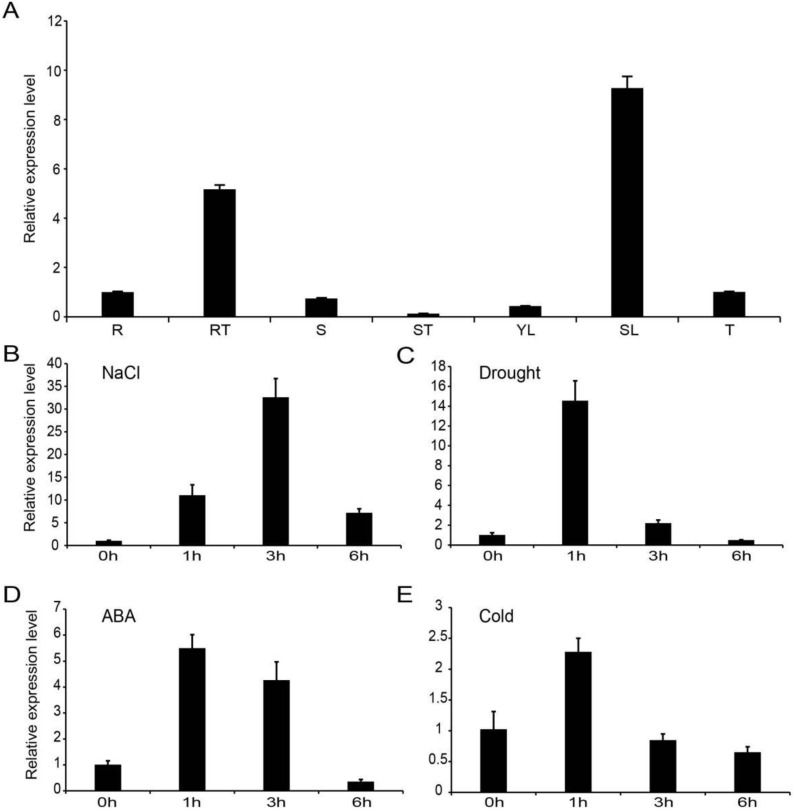
The expression pattern of *StNAC053* in potato. (**A**) The expression profiles of *StNAC053* in seven tissues. R: root; RT: root tip; S: stem; ST: stem tip; YL: young leaf; SL: senescent leaf; T: tuber. (**B**–**E**) The expression pattern of *StNAC053* under salt, drought, ABA, and cold treatments. Whole plants of treated potato seedlings were collected for qRT-PCR assays.

To further investigate the gene expression pattern of *StNAC053*, qRT-PCR was conducted to detect the transcription levels of *StNAC053* in different tissue types and in response to various abiotic stresses. The results showed that *StNAC053* had relatively high expression levels in roots, root tips, stems, tubers, and the highest expression level was observed in senescent leaves. *StNAC053* expression was relatively low in young leaves and stem tips (Figure 3A). Whole plants of treated potato seedlings were collected for qRT-PCR assays. Under salt stress conditions, the expression of *StNAC053* was induced during 1–6 h after treatments and reached peak levels with a 32.6-fold increase at 3 h (Figure 3B). *StNAC053* transcript was upregulated specifically at 1 and 3 h after drought stress and ABA treatments, and reached peak levels with 14.5- and 5.5-fold increases, respectively, at 1 h (Figure 3C,D). The cold stress treatments induced *StNAC053* expression within 1 h, and then the expression declined until 6 h (Figure 3E). These results indicated that the expression of *StNAC053* can be induced by salt and drought stresses. Promoter analysis showed that the *StNAC053* promoter contains one MYB binding site (MBS) that is related to drought inducibility, one stress-responsive element (TC-rich repeats), and four elements related to ABA responsiveness (ABRE), suggesting that the StNAC053 might be involved in plants’ response to ABA-mediated abiotic stresses (Supplementary Figure S2).

### 2.4. Subcellular Location and Transactivation Activity Assay

**Figure 4:**
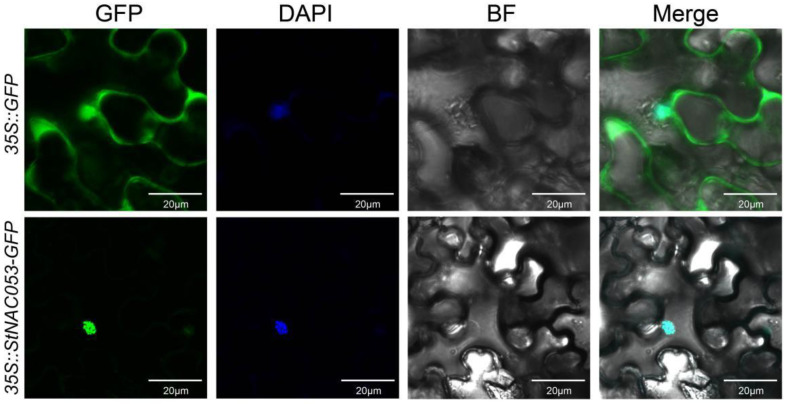
The subcellular localization of StNAC053 in *N. benthamiana* epidermal cells. GFP and *StNAC053-GFP* fusion construct under the control of *CaMV-35S* promoter were transiently expressed into *N. benthamiana* epidermal cells, respectively. Bar = 20 μm, DAPI, 4,6-diamidino-2-phenylindole for nuclear staining.

In order to analyze the subcellular localization of the StNAC053 protein, the *35S::GFP-StNAC053* fusion vector and *35S::GFP* empty vector (control) were transiently expressed in *N. benthamiana* and the subcellular localization of the GFP signals was observed by confocal microscopy. The green fluorescence of the StNAC053-GFP fusion proteins was only observed in the nucleus, whereas the signals of the control were distributed on plasma membranes and in the cytoplasm and nucleus (Figure 4). These results demonstrated that the StNAC053 protein was located in the nucleus.

**Figure 5:**
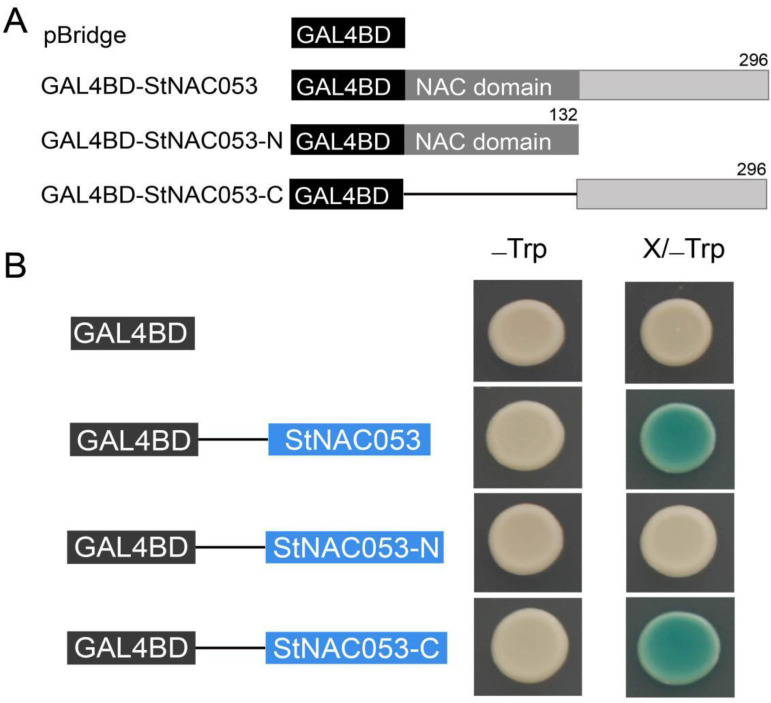
Transactivation assay of StNAC053 in yeasts. (**A**) Different truncations of *StNAC053* fused to the *GAL4* DNA binding domain in pBridge vector. (**B**) Transactivation activity assay of StNAC053 in yeast strain AH109. β-galactosidase activity against x-gal was detected on the SD/-Trp medium.

To investigate the transcriptional activation ability of StNAC053, the full length CDS, N-terminal region, and C-terminal region of the coding sequence were inserted into the pBridge vector, respectively (Figure 5A). The three constructs and pBridge empty vector (control) were then transformed into the yeast strain AH109, and all transformed yeast cells grew well on the SD/-Trp medium. Subsequently, these well-grown yeast cells were transferred from SD/-Trp media to SD/-Trp/x-gal media. Activation of the reporter gene in the yeast cells was determined by assays of the β-galactosidase activity with x-gal as substrate. The yeast strain containing the full-length StNAC053 (GAL4BD-StNAC053) and the C-terminus of StNAC053 (GAL4BD-StNAC053C) were blue on the SD/-Trp/x-gal medium, whereas the cells with the N-terminus of StNAC053 (GAL4BD-StNAC053N) and the pBridge empty vector were not blue (Figure 5B). The above described results showed that StNAC053 is a transcriptional activator, and its transactivation domain is in the C-terminus.

### 2.5. StNAC053 Overexpression in Arabidopsis Confers ABA Hypersensitivity

In order to further study the roles of *StNAC053* in response to salt and drought stresses, transgenic *Arabidopsis* plants overexpressing *StNAC053* were generated. Eight T0 transgenic lines were verified by normal PCR. The homozygous T3 transgenic lines were selected, and the expression level of *StNAC053* was analyzed using qRT-PCR. Two overexpression lines (*OE2* and *OE6*) with higher expression levels of *StNAC053* were selected for further phenotypic analysis (Supplementary Figure S3).

**Figure 6:**
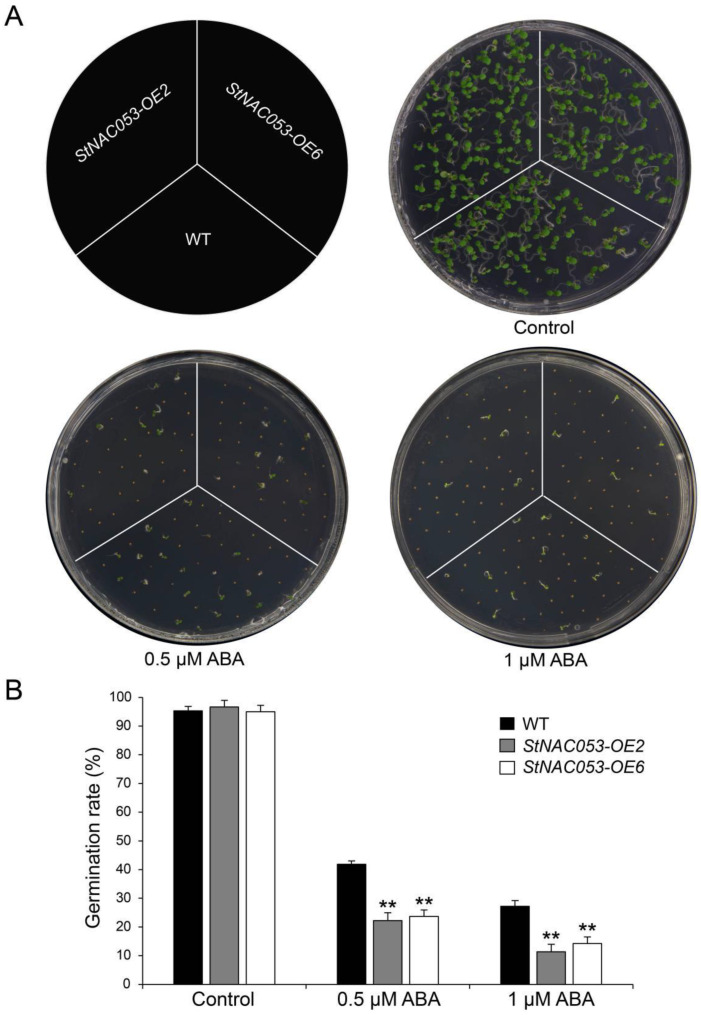
Comparison of the ABA sensitivity between *StNAC053* overexpression and wild-type *Arabidopsis*. (**A**) Seed germination assay between *StNAC053* overexpression and wild-type *Arabidopsis* under 0.5 and 1 μM ABA treatments. The seed germination rates were recorded six days after sowing. (**B**) The statistics of germination rates under normal condition, 0.5 and 1 μM ABA treatments. WT, wild-type. The data were means ± SD from three independent replications. ** p < 0.01 (*t*-tests).

As described above, expression of *StNAC053* in potato seedlings was upregulated within 3 h after ABA treatment and reached a peak at 1 h (Figure 3D). In order to find out whether StNAC053 is involved in ABA response, we conducted a germination assay to study the sensitivity of *StNAC053-OE* lines to ABA treatments. The results showed that without ABA treatment, the germination rates were almost identical between the *StNAC053-OE* lines and WT. However, when grown on half-MS media containing 0.5 or 1.0 μM ABA, the germination rates of both *StNAC053-OE* lines and WT reduced significantly, while germination rates of the *StNAC053-OE* lines were significantly lower than that of WT (Figure 6A), indicating that *StNAC053-OE* plants were more sensitive to exogenous ABA in the seed germination assay (Figure 6B).

### 2.6. StNAC053 Overexpression Enhances Salt Tolerance in Transgenic Arabidopsis

**Figure 7:**
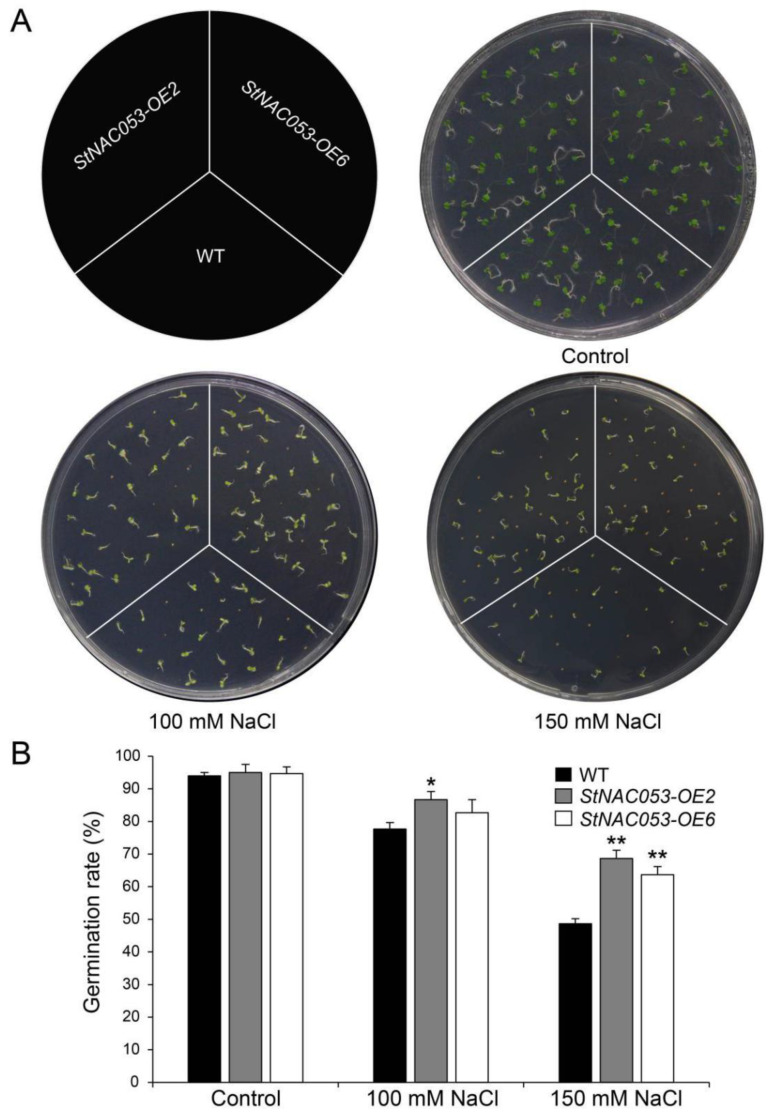
Comparison of seed germination between *StNAC053* overexpressing and wild-type *Arabidopsis* under salt stress treatments. (**A**) Seed germination assay between *StNAC053* overexpression and wild-type *Arabidopsis* under 100 and 150 mM NaCl treatments. The seed germination rates were recorded six days after sowing. (**B**) The statistics of seed germination under normal condition, 100 and 150 mM NaCl treatments. WT, wild-type. The data were means ± SD from three independent replications. * *p* < 0.05, ** *p* < 0.01 (*t*-tests).

Germination assays were also used to investigate the salt tolerance of *StNAC053-OE* lines. As a result, the *StNAC053-OE* lines displayed higher germination rates than WT on half-MS media containing 100 and 150 mM NaCl (Figure 7).

**Figure 8:**
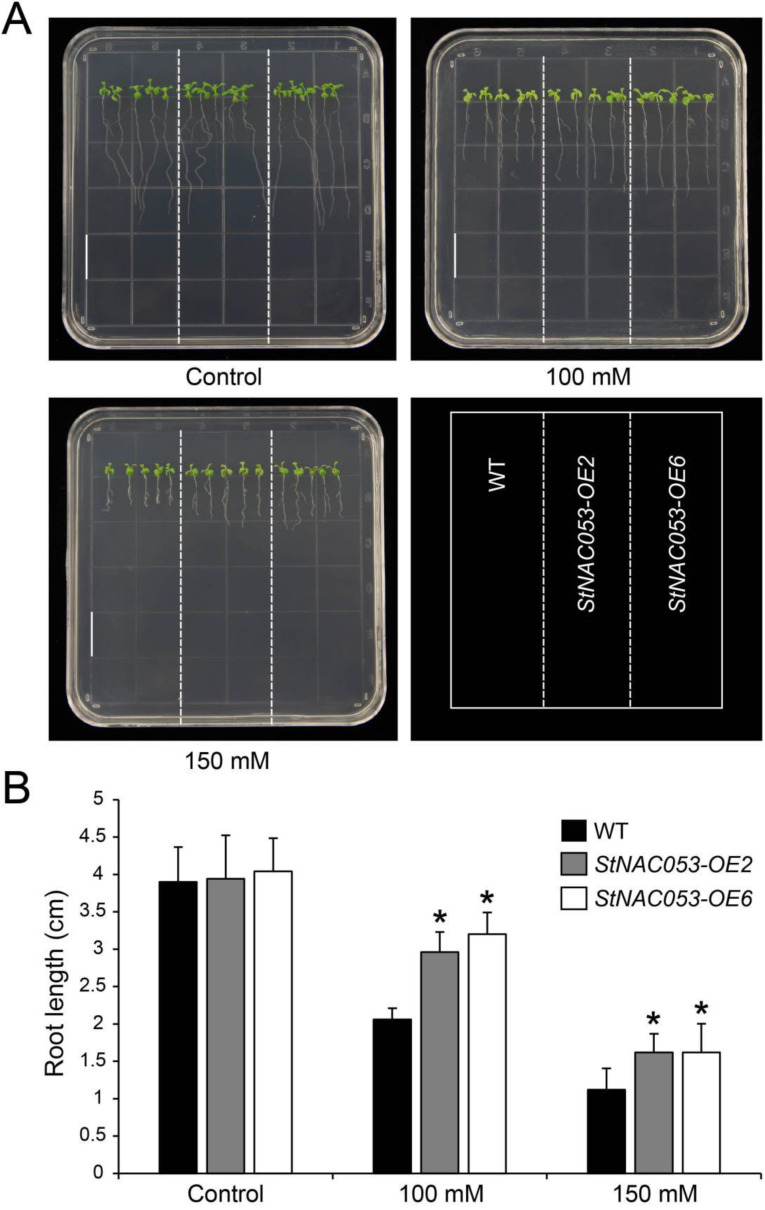
Comparison of root growth between *StNAC053* overexpressing and wild-type *Arabidopsis* under salt stress treatments. (**A**) The primary root length of *StNAC053* overexpressing and wild-type *Arabidopsis* under 100 and 150 mM NaCl treatments. The primary root length were recorded seven days after growth. Bar = 1.5 cm. (**B**) The statistics of primary root length under normal condition, 100 and 150 mM NaCl treatments. WT, wild-type. The data were means ± SD from three independent replications. * *p* < 0.05 (*t*-tests).

In addition, a root elongation assay was conducted to further determine the response of the *StNAC053-OE* lines to salt stress. Seven days after growing on half-MS media containing 100 or 150 mM NaCl, primary roots of the *StNAC053-OE* lines were found to be significantly longer compared to that of the WT (Figure 8). Taken together, results from the salt treatment assays suggested that overexpression of *StNAC053* improved the tolerance to salt stress in transgenic *Arabidopsis* plants.

### 2.7. StNAC053 Overexpression Improves Drought Tolerance in Transgenic Arabidopsis

**Figure 9:**
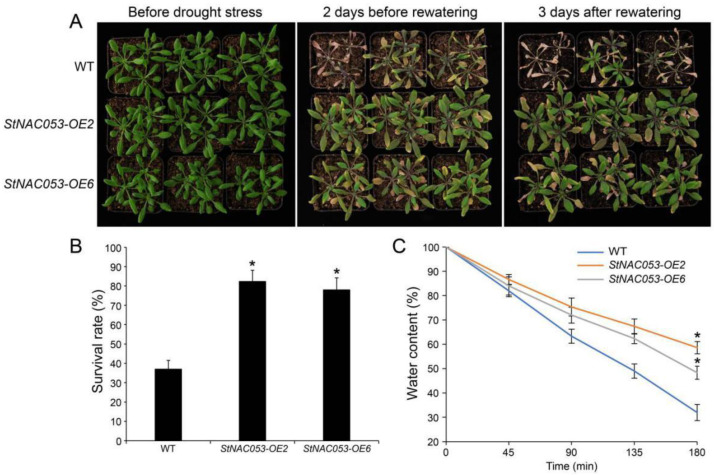
Transgenic *StNAC053 Arabidopsis* showed higher drought tolerance. (**A**) Phenotypes of *StNAC053* overexpressing and wild-type *Arabidopsis* under drought stress. The *StNAC053* overexpressing and wild-type *Arabidopsis* were grown for four weeks under normal conditions. Watering was ceased for two weeks, followed by rewatering for recovery. (**B**) Statistical analysis of survival rates after three days for rewatering. (**C**) Water loss rates of detached rosette leaves of *StNAC053* overexpressing and wild-type *Arabidopsis*. WT, wild-type. The data were means ± SD from three independent replications. * *p* < 0.05 (*t*-tests).

To study the role of StNAC053 in drought response, one-week-old seedling of *StNAC053-OE2*, *StNAC053-OE6*, and WT were transferred from half-MS media to water-saturated matrix and grown for four weeks. Subsequently, these plants were treated with drought stress via water withholding. After two weeks of drought treatments, while most of the WT plants wilted, the *StNAC053-OE* plants displayed slight leaf rolling (Figure 9A). After rewatering for three days, only 37% of the WT plants were recovered, whereas 82% and 78% of plants of the two *StNAC053-OE* lines survived, respectively (Figure 9B). Furthermore, the *StNAC053-OE* plants showed higher water content than WT plants during the 3 h dehydration (Figure 9C). These results suggested that the *StNAC053* overexpression improved the tolerance of the *Arabidopsis* plants to drought stress.

### 2.8. StNAC053 Overexpression Enhances ROS-Scavenging Capability in Transgenic Arabidopsis

**Figure 10:**
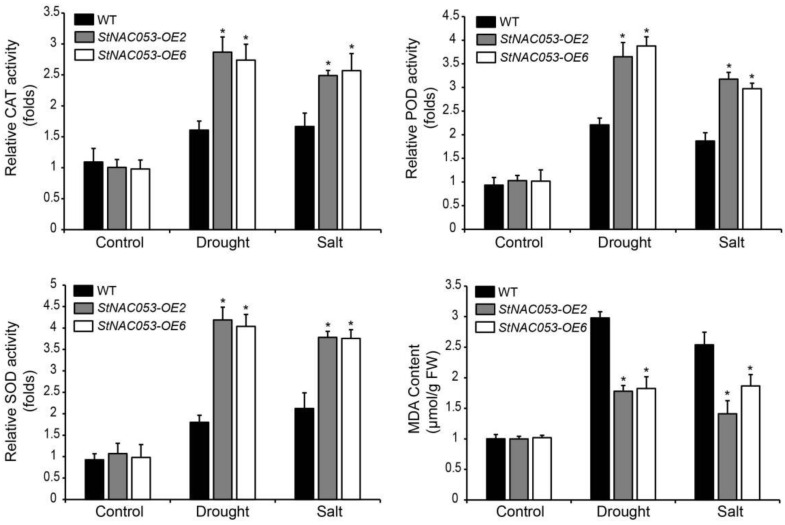
Changes of antioxidant enzyme activities and MDA contents in *StNAC053* overexpressing and wild-type *Arabidopsis* after treatments with drought or salt stress. MDA, malonic dialdehyde; CAT, catalase; POD, peroxidase; SOD; superoxide. WT, wild-type. The data were means ± SD from three independent replications. * *p* < 0.05.

Changes in reactive oxygen species (ROS) are often associated with plants’ response to abiotic stresses. In order to determine whether changes of antioxidant capacity are associated with increased tolerance to salt and drought stresses of the *StNAC053-OE* plants, we measured the stress-related physiological parameters of *StNAC053-OE* lines and WT plants with or without stress treatments for three days (Figure 10). The activities of the ROS-scavenging enzymes CAT (catalase), POD (peroxidase), and SOD (superoxide) were comparable between the *StNAC053-OE* lines and WT without drought or salt stress. When subjected to salt or drought stress, the activities of CAT, POD, and SOD in plants of *StNAC053-OE* lines and WT were all increased. However, significantly more increase of ROS-scavenging activities was observed in the *StNAC053-OE* lines than in WT. Moreover, there was no significant difference in MDA (malonic dialdehyde) contents between the *StNAC053-OE* lines and WT without stress treatments. However, significantly lower MDA levels were detected in *StNAC053-OE* plants when subjected to salt or drought stress. These results suggested that *StNAC053* transgenic *Arabidopsis* plants might have enhanced stress tolerance due to increased ROS-scavenging capability.

### 2.9. StNAC053 Regulates the Expression of Stress-Responsive Genes

**Figure 11:**
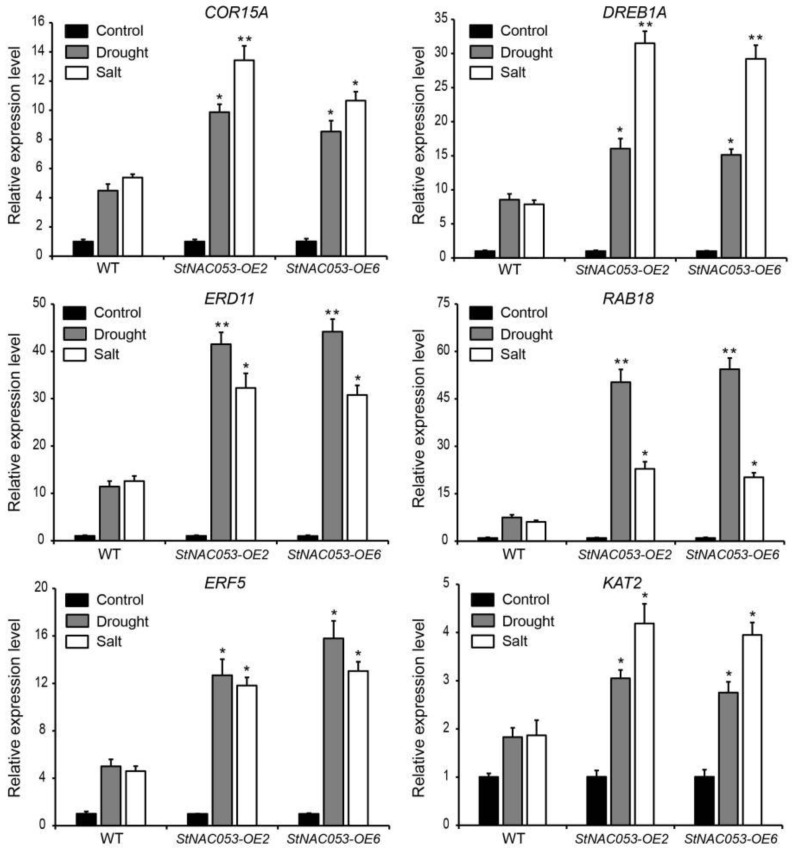
Expression of stress-responsive genes in *StNAC053* overexpressing and wild-type *Arabidopsis* after drought or salt stress treatments. WT, wild-type. The data were means ± SD from three independent replications. * *p* < 0.05, ** *p*< 0.01.

qRT-PCR was preformed to examine whether StNAC053 regulates salt and drought responses through regulating expression of stress-responsive genes. There was no significant difference in the expression of selected stress-responsive genes between *StNAC053-OE* and WT plants under normal growth conditions. When subjected to drought or salt stress, the expression of the stress-responsive genes was upregulated in both *StNAC053-OE* and WT plants. However, compared to the WT, the expression levels of the stress-responsive genes were significantly higher in the *StNAC053-OE* lines (Figure 11). These results suggested that *StNAC053* transgenic plants might enhance drought or salt tolerance by activating the expression of stress-responsive genes.

## 3. Discussion

The NAC transcription factors comprise one of the largest transcription factor families in plants and play multiple roles in stress responses. In a previous study, a total of 110 potato NAC family members were identified and several of them were identified to be highly induced by various stress treatments [21]. A number of *NAC* genes has been characterized and analyzed in different plant species, including *Arabidopsis* and rice. The yield of potato could be severely affected by various abiotic stresses, especially salt and drought. However, little information is available on the biological functions of the potato *NAC* genes. In the present study, an NAC transcription factor was cloned from potato, which possessed a typical NAC structure and was classified in the ATAF subgroup (Figure 1 and Figure 2A).

In plants, a number of NAC transcriptional factors have been identified to be involved in ABA-mediated abiotic stress responses [40,41]. In this study, we found that the promoter region of *StNAC053* possessed four ABRE motifs, which has been known as an important *cis*-element involved in ABA signaling (Supplementary Figure S2). The results from expression analysis revealed that *StNAC053* was significantly induced by ABA treatments (Figure 3D). Furthermore, seed germination rates of *StNAC053-OE* lines were significantly lower than those of WT plants, suggesting that overexpression of *StNAC053* caused transgenic plants to be hypersensitive to ABA (Figure 6).

Multiple NAC transcription factors have been reported to be involved in plants’ responses to abiotic stresses, including salt, drought, heat, and cold treatments [29,42]. In potato, *StNAC053* was found to be induced by various abiotic stress treatments, especially drought and salt stresses, implying that StNAC053 may play important roles in potato’s response to drought and salt stresses (Figure 3). In this study, we focused on the role of StNAC053 in salt and drought stress responses. Under high salinity conditions, the germination rates and root lengths of *StNAC053*-*OE* plants were significantly higher than those of WT (Figure 7 and Figure 8). In addition, *StNAC053-OE* plants had higher survival rates than WT plants under drought conditions (Figure 9). Thus, our results showed that *StNAC053*-*OE Arabidopsis* transgenic plants had significantly enhanced salt and drought tolerance. Similarly, overexpression of the *StNAC053* homolog *ATAF1* conferred drought tolerance in *Arabidopsis* [43]. Additionally, under drought or salt stress, a large amount of ROS were produced. Excessive ROS could damage the cellular membrane [44], resulting in the generation of massive secondary products such as MDA [45]. The antioxidant enzymes played an important role in coping with excess ROS to reduce oxidative stress. CAT, POD, and SOD are among the three most important antioxidant enzymes [44]. After drought or salt stress, the levels of MDA in *StNAC053-OE* lines were significantly decreased compared with WT, suggesting that *StNAC053-OE* lines suffered less oxidative damage (Figure 10). Meanwhile, the enzyme activities of CAT, POD, and SOD were significantly higher, suggesting that *StNAC053-OE* lines have higher ROS-scavenging capacity (Figure 10). Notably, although growth retardation is usually accompanied with the overexpression of NAC transcription factors [46,47], no significant developmental difference was observed between the transgenic plants and WT in this study.

Most of the NAC transcription factors were involved in stress responses function as transcription activators in regulating downstream stress-responsive genes [48]. AtJUB1 can directly activate the transcription of *AtDREB2A* in *Arabidopsis* [49]. Rice ONAC066 enhanced drought tolerance through regulating the expression of *OsDREB2A* [50]. In the current study, StNAC053 was identified to function as a transcription activator (Figure 4 and Figure 5) and positively regulate the expression of multiple stress-responsive genes such as *COR15A*, *DREB1A*, *KAT2*, etc. (Figure 11). These genes encode stress-associated proteins that act as key mediators in protecting plants from oxidative or osmotic damages. The promoter regions of all these genes were found to harbor NAC binding sites (Supplementary Table S1) to which the StNAC053 transcription factor might bind when activating the expression of these stress-responsive genes under stress conditions. StNAC053 holds a promising utility as an excellent candidate in the genetic improvement of potato abiotic stress tolerance because it positively regulates plant stress tolerance but does not have any negative influence on plant growth.

## 4. Materials and Methods

### 4.1. Potato Plant Preparation and Treatments

Potato cultivar GN2 was used to analyze the expression of *StNAC053* gene in this study. Potato sprouts were incubated on complete MS solid media by nodule cutting and cultivated in a growth chamber at 24 °C under continuous light. For stress treatments, seedlings were treated with dehydration, at 4 °C, with NaCl (200 mM), and ABA (50 μM) for 0, 1, 3, and 6 h, respectively. All of the harvested samples were immediately frozen in liquid nitrogen, and stored at −80 °C, prior to RNA extraction. Three biological replicates were used for each sample. The *Arabidopsis thaliana* Col-0 plants were used for transgenic study of *StNAC053*.

### 4.2. RNA Extraction and qRT-PCR

Total RNAs of the tested samples were extracted using the Ultrapure RNA Kit (cwbiotech, Beijing, China), and the first-strand complementary DNA (cDNA) was synthesized using the PrimeScript™ RT reagent Kit (TaKaRa, Dalian, China). Expression of six stress-responsive genes from *Arabidopsis* including *COR15A* (U01377.1), *DREB1A* (AB013815.1), *ERD11* (D17672.1), *RAB18* (X68042.1), *ERF5* (NM_124094.3), and *KAT2* (NM_001341273.1) was determined. The potato *EF1α* gene and *Arabidopsis Actin 2* gene were used as internal controls [21,39], and quantitative real-time PCR (qRT-PCR) reactions were performed with 40 cycles in a Roche LightCycler 480 Real-Time PCR instrument. All expression data were obtained from three technical repeats, and calculated by the 2^−^^△△^^CT^ method. The primer sequences used in the current study are listed in Supplementary Table S2.

### 4.3. Cloning and Sequence Analysis of StNAC053

The coding sequence of *StNAC053* was amplified using RT-PCR. The purified PCR products were inserted into the subclone vector pEASY-Blunt, and the constructed vector was then transformed into *E.coli* trans T1 competent cells (Trans Gen, Beijing, China). After sequencing, the recombinant plasmid was named Blunt-StNAC053. The ProtParam online tool (https://web.expasy.org/protparam/) (accessed on 20 January 2020) was used to analyze the molecular weight, isoelectric point of the StNAC053 protein. The NLS of NAC proteins was predicted via cNLS Mapper (http://nls-mapper.iab.keio.ac.jp) (accessed on 20 January 2020). Multiple sequence alignment was carried out using the DNAMAN tool. A neighbor-joining tree was generated using MEGA6 with 1000 replications. The NAC protein sequences were obtained from the TAIR (https://www.arabidopsis.org/) (accessed on 20 January 2020), SGN (http://solgenomics.net/) (accessed on 20 January 2020), and NCBI. The conserved motifs of NAC protein sequences were analyzed via MEME (http://meme-suite.org/) (accessed on 20 January 2020) with default parameters. To assess the promoter *cis*-acting elements of the *StNAC053* gene, 2000 bp of promoter regions upstream of the start codon of the *StNAC053* gene were extracted. PlantCARE (http://bioinformatics.psb.ugent.be/webtools/plantcare/html/) (accessed on 20 January 2020) was used for *cis*-acting regulatory element investigation.

### 4.4. Subcellular Localization

The coding sequence (without stop codon) of *StNAC053* was amplified using PCR. The purified PCR products were inserted into the pCHF3-cGFP vector at the speI site by Infusion (Clontech), yielding plasmid 35S::StNAC053-GFP after sequencing. The construct was then transformed into *Agrobacterium* GV3101competent cells, and transiently expressed in the leaves of *Nicotiana benthamiana* [51]. Simultaneously, the empty pCHF3-cGFP vector was used as a control. Three days after injection, the leaves were soaked in the DAPI (dye 4,6-diamidino-2-phenylindole) staining solution and observed under a confocal laser microscope (TCS-SP8 Leica, Wetzlar, Germany) to detect fluorescence signals of the fusion protein, as previously reported [52].

### 4.5. Transactivation Assay

For transactivation activity assay of StNAC053, the full length CDS and two truncated sequences of *StNAC053* genes were amplified using PCR and inserted into the pBridge vector at the *EcoR*I site by Infusion (Clontech, Bejing, China) to fuse with a GAL4 DNA binding domain, yielding plasmid pBridge-StNAC053. Plasmid pBridge-StNAC053 and empty pBridge vector (control) were then transformed into the yeast strain AH109, respectively. The transformed yeasts were plated on SD media lacking tryptophan (SD/-Trp) and incubated at 30 °C for 3 days. Subsequently, the positive clones were transferred to SD/-Trp media supplemented with x-gal and incubated at 30 °C for 3 days. Transactivation activity of the fused proteins was determined based on the growth status (blue/white) of the transformants.

### 4.6. Generation of StNAC053 Transgenic Arabidopsis Plants

For overexpression analysis, the full-length CDS of *StNAC053* was amplified using PCR and inserted into the pCHF3 vector at the *Sac*I site by Infusion (Clontech, Bejing, China), yielding plasmid 35S::StNAC053 after sequencing. The construct was then transformed into *Agrobacterium* GV3101 competent cells. The positive colonies of *Agrobacterium* were then selected and used in transforming *Arabidopsis* Col-0 plants by the floral dip method [53]. The T0 generation seeds were screened by half-strength MS media with 50 mg/L kanamycin to obtain *StNAC053* overexpression plants. T3 homozygous lines were selected for further phenotypic analysis.

### 4.7. Seedling Growth Assays

For seed germination assays, *Arabidopsis* seeds were firstly sterilized using 75% alcohol and then evenly sown on half-MS media containing ABA (0.5 and 1.0 μM) or NaCl (100 and 150 mM), respectively. Seeds were stratified at 4 °C for 2 days under darkness, and then moved to a growth chamber (23 °C, continuous light). After cultivation for 6 days, the germination rates were calculated with three replications.

For root elongation assays, *Arabidopsis* seedlings were grown normally on half-MS media for 7 days. Seedlings with similar growth status were then transferred to half-MS media containing 100 and 150 mM NaCl, respectively. The length of the primary root was measured after 7 days with three replications.

### 4.8. Drought Stress Tolerance Assays of StNAC053 Transgenic Arabidopsis Plants

For the drought tolerance assays, one-week-old seedlings germinated on half-MS media were transferred into pots containing soil mixture (peat moss and vermiculite, 3:1 *v*/*v*). Four-week-old plants grown under normal conditions were exposed to drought stress for 14 days. The plants were then rewatered for 3 days, and the survival rates were recorded. The detached leaves were air-dried for 3 h, and were weighed at 5 time points (0, 45, 90, 135, and 180 min). The rate of water loss was calculated based on the weight loss at each time point divided by the initial fresh weight.

### 4.9. Physiological Measurements

The physiological parameters were measured using fully expanded leaves obtained from well-watered, drought-stressed, or salt-stressed plants. The content of MDA and the enzymatic activities of superoxide (SOD), peroxidase (POD), and catalase (CAT) were determined as previously described [54]. Three biological replicates were performed.

### 4.10. Statistical Analysis

The statistical significance was analyzed using the GraphPad Prism 8 *t* test. *p* values less than 0.05 or 0.01 were regarded as significantly different from the control. All data were obtained from three replicates.

## 5. Conclusions

In this study, a typical NAC transcription factor StNAC053 was cloned and identified to be a transcription activator. The *Arabidopsis* plants overexpressing *StNAC053* displayed significantly increased tolerance to salt and drought stress treatments, while the overexpression lines seem to be more sensitive to ABA treatments. Taken together, the transcription factor StNAC053 might be involved in drought and salt stress responses via ABA-mediated signaling in potato.

## Data Availability

Data is contained within the article or supplementary material.

## Supplementary Materials

The following are available online at https://www.mdpi.com/1422-0067/22/5/2568/s1, Figure S1: The putative conserved motifs in NAC proteins, Figure S2: Stress-related elements in the promoter regions of *StNAC053* gene, Figure S3: The expression level of the *StNAC053* gene in wild-type and two overexpression lines, the ratios of gene expression levels were calculated relative to the wild-type, ** *p*< 0.01, Table S1: Prediction of NAC binding *cis*-elements in the promoter of stress-responsive genes, Table S2: The primers used in this study.

## References

[1] Umezawa T., Fujita M., Fujita Y., Yamaguchi-Shinozaki K., Shinozaki K. (2006). Engineering drought tolerance in plants: Discovering and tailoring genes to unlock the future. Curr. Opin. Biotechnol..

[2] Shinozaki K., Yamaguchi-Shinozaki K., Seki M. (2003). Regulatory network of gene expression in the drought and cold stress responses. Curr. Opin. Plant Biol..

[3] Fujita M., Fujita Y., Maruyama K., Seki M., Hiratsu K., Ohme-Takagi M., Tran L.S., Yamaguchi-Shinozaki K., Shinozaki K. (2004). A dehydration-induced NAC protein, RD26, is involved in a novel ABA-dependent stress-signaling pathway. Plant J..

[4] Chen L., Song Y., Li S., Zhang L., Zou C., Yu D. (2012). The role of WRKY transcription factors in plant abiotic stresses. Biochim. Biophys. Acta.

[5] Guo H., Wang Y., Wang L., Hu P., Wang Y., Jia Y., Zhang C., Zhang Y., Zhang Y., Wang C. (2017). Expression of the MYB transcription factor gene BplMYB46 affects abiotic stress tolerance and secondary cell wall deposition in *Betula platyphylla*. Plant Biotechnol. J..

[6] Guo Y., Gan S. (2006). AtNAP, a NAC family transcription factor, has an important role in leaf senescence. Plant J..

[7] Kim Y.S., Sakuraba Y., Han S.H., Yoo S.C., Paek N.C. (2013). Mutation of the *Arabidopsis* NAC016 transcription factor delays leaf senescence. Plant. Cell Physiol..

[8] Mitsuda N., Iwase A., Yamamoto H., Yoshida M., Seki M., Shinozaki K., Ohme-Takagi M. (2007). NAC transcription factors, NST1 and NST3, are key regulators of the formation of secondary walls in woody tissues of *Arabidopsis*. Plant Cell.

[9] Zhang J., Huang G.Q., Zou D., Yan J.Q., Li Y., Hu S., Li X.B. (2018). The cotton (*Gossypium hirsutum*) NAC transcription factor (FSN1) as a positive regulator participates in controlling secondary cell wall biosynthesis and modification of fibers. New Phytol..

[10] Xie Q., Frugis G., Colgan D., Chua N.H. (2000). *Arabidopsis* NAC1 transduces auxin signal downstream of TIR1 to promote lateral root development. Genes Dev..

[11] He X.J., Mu R.L., Cao W.H., Zhang Z.G., Zhang J.S., Chen S.Y. (2005). AtNAC2, a transcription factor downstream of ethylene and auxin signaling pathways, is involved in salt stress response and lateral root development. Plant J..

[12] Zhang L., Yao L., Zhang N., Yang J., Zhu X., Tang X., Calderon-Urrea A., Si H. (2018). Lateral Root Development in Potato Is Mediated by Stu-mi164 Regulation of NAC Transcription Factor. Front. Plant Sci..

[13] Takada S., Hibara K., Ishida T., Tasaka M. (2001). The CUP-SHAPED COTYLEDON1 gene of *Arabidopsis* regulates shoot apical meristem formation. Development.

[14] Larsson E., Sundstrom J.F., Sitbon F., von Arnold S. (2012). Expression of PaNAC01, a Picea abies CUP-SHAPED COTYLEDON orthologue, is regulated by polar auxin transport and associated with differentiation of the shoot apical meristem and formation of separated cotyledons. Ann. Bot..

[15] Hendelman A., Stav R., Zemach H., Arazi T. (2013). The tomato NAC transcription factor SlNAM2 is involved in flower-boundary morphogenesis. J. Exp. Bot..

[16] Chen X., Lu S., Wang Y., Zhang X., Lv B., Luo L., Xi D., Shen J., Ma H., Ming F. (2015). OsNAC2 encoding a NAC transcription factor that affects plant height through mediating the gibberellic acid pathway in rice. Plant J..

[17] Shahnejat-Bushehri S., Tarkowska D., Sakuraba Y., Balazadeh S. (2016). *Arabidopsis* NAC transcription factor JUB1 regulates GA/BR metabolism and signalling. Nat. Plants.

[18] Cao L., Yu Y., Ding X., Zhu D., Yang F., Liu B., Sun X., Duan X., Yin K., Zhu Y. (2017). The Glycine soja NAC transcription factor GsNAC019 mediates the regulation of plant alkaline tolerance and ABA sensitivity. Plant Mol. Biol..

[19] Kim Y.S., Kim S.G., Park J.E., Park H.Y., Lim M.H., Chua N.H., Park C.M. (2006). A membrane-bound NAC transcription factor regulates cell division in *Arabidopsis*. Plant Cell.

[20] Nuruzzaman M., Manimekalai R., Sharoni A.M., Satoh K., Kondoh H., Ooka H., Kikuchi S. (2010). Genome-wide analysis of NAC transcription factor family in rice. Gene.

[21] Singh A.K., Sharma V., Pal A.K., Acharya V., Ahuja P.S. (2013). Genome-wide organization and expression profiling of the NAC transcription factor family in potato (*Solanum tuberosum* L.). DNA Res..

[22] Saidi M.N., Mergby D., Brini F. (2017). Identification and expression analysis of the NAC transcription factor family in durum wheat (*Triticum turgidum* L. ssp. durum). Plant Physiol. Biochem..

[23] Rushton P.J., Bokowiec M.T., Han S., Zhang H., Brannock J.F., Chen X., Laudeman T.W., Timko M.P. (2008). Tobacco transcription factors: Novel insights into transcriptional regulation in the Solanaceae. Plant Physiol..

[24] Le D.T., Nishiyama R., Watanabe Y., Mochida K., Yamaguchi-Shinozaki K., Shinozaki K., Tran L.S. (2011). Genome-wide survey and expression analysis of the plant-specific NAC transcription factor family in soybean during development and dehydration stress. DNA Res..

[25] Wei S., Gao L., Zhang Y., Zhang F., Yang X., Huang D. (2016). Genome-wide investigation of the NAC transcription factor family in melon (*Cucumis melo* L.) and their expression analysis under salt stress. Plant Cell Rep..

[26] Tran L.S., Nakashima K., Sakuma Y., Simpson S.D., Fujita Y., Maruyama K., Fujita M., Seki M., Shinozaki K., Yamaguchi-Shinozaki K. (2004). Isolation and functional analysis of *Arabidopsis* stress-inducible NAC transcription factors that bind to a drought-responsive cis-element in the early responsive to dehydration stress 1 promoter. Plant Cell.

[27] He L., Shi X., Wang Y., Guo Y., Yang K., Wang Y. (2017). *Arabidopsis* ANAC069 binds to C[A/G]CG[T/G] sequences to negatively regulate salt and osmotic stress tolerance. Plant Mol. Biol..

[28] Seok H.Y., Woo D.H., Nguyen L.V., Tran H.T., Tarte V.N., Mehdi S.M., Lee S.Y., Moon Y.H. (2017). *Arabidopsis* AtNAP functions as a negative regulator via repression of AREB1 in salt stress response. Planta.

[29] Fang Y., Liao K., Du H., Xu Y., Song H., Li X., Xiong L. (2015). A stress-responsive NAC transcription factor SNAC3 confers heat and drought tolerance through modulation of reactive oxygen species in rice. J. Exp. Bot..

[30] Jeong J.S., Kim Y.S., Baek K.H., Jung H., Ha S.H., Do Choi Y., Kim M., Reuzeau C., Kim J.K. (2010). Root-specific expression of OsNAC10 improves drought tolerance and grain yield in rice under field drought conditions. Plant Physiol..

[31] Hong Y., Zhang H., Huang L., Li D., Song F. (2016). Overexpression of a Stress-Responsive NAC Transcription Factor Gene ONAC022 Improves Drought and Salt Tolerance in Rice. Front. Plant Sci..

[32] Hao Y.J., Wei W., Song Q.X., Chen H.W., Zhang Y.Q., Wang F., Zou H.F., Lei G., Tian A.G., Zhang W.K. (2011). Soybean NAC transcription factors promote abiotic stress tolerance and lateral root formation in transgenic plants. Plant J..

[33] Mao X., Zhang H., Qian X., Li A., Zhao G., Jing R. (2012). TaNAC2, a NAC-type wheat transcription factor conferring enhanced multiple abiotic stress tolerances in *Arabidopsis*. J. Exp. Bot..

[34] Xia N., Zhang G., Liu X.Y., Deng L., Cai G.L., Zhang Y., Wang X.J., Zhao J., Huang L.L., Kang Z.S. (2010). Characterization of a novel wheat NAC transcription factor gene involved in defense response against stripe rust pathogen infection and abiotic stresses. Mol. Biol. Rep..

[35] Xia N., Zhang G., Sun Y.F., Zhu L., Xu L.S., Chen X.M., Liu B., Yu Y.T., Wang X.J., Huang L.L. (2010). TaNAC8, a novel NAC transcription factor gene in wheat, responds to stripe rust pathogen infection and abiotic stresses. Physiol. Mol. Plant Pathol..

[36] Zhang Z.L., Liu C., Guo Y.F. (2020). Wheat Transcription Factor TaSNAC11-4B Positively Regulates Leaf Senescence through Promoting ROS Production in Transgenic *Arabidopsis*. Int. J. Mol. Sci..

[37] Mao H., Wang H., Liu S., Li Z., Yang X., Yan J., Li J., Tran L.S., Qin F. (2015). A transposable element in a NAC gene is associated with drought tolerance in maize seedlings. Nat. Commun..

[38] Mao H., Yu L., Han R., Li Z., Liu H. (2016). ZmNAC55, a maize stress-responsive NAC transcription factor, confers drought resistance in transgenic *Arabidopsis*. Plant Physiol. Biochem..

[39] Lu M., Ying S., Zhang D.F., Shi Y.S., Song Y.C., Wang T.Y., Li Y. (2012). A maize stress-responsive NAC transcription factor, ZmSNAC1, confers enhanced tolerance to dehydration in transgenic *Arabidopsis*. Plant Cell Rep..

[40] Nakashima K., Takasaki H., Mizoi J., Shinozaki K., Yamaguchi-Shinozaki K. (2012). NAC transcription factors in plant abiotic stress responses. Biochim. Biophys. Acta.

[41] Shao H., Wang H., Tang X. (2015). NAC transcription factors in plant multiple abiotic stress responses: Progress and prospects. Front. Plant Sci..

[42] Iuchi S., Kobayashi M., Taji T., Naramoto M., Seki M., Kato T., Tabata S., Kakubari Y., Yamaguchi-Shinozaki K., Shinozaki K. (2001). Regulation of drought tolerance by gene manipulation of 9-cis-epoxycarotenoid dioxygenase, a key enzyme in abscisic acid biosynthesis in *Arabidopsis*. Plant J..

[43] Wu Y., Deng Z., Lai J., Zhang Y., Yang C., Yin B., Zhao Q., Zhang L., Li Y., Yang C. (2009). Dual function of *Arabidopsis* ATAF1 in abiotic and biotic stress responses. Cell Res..

[44] Gill S.S., Tuteja N. (2010). Reactive oxygen species and antioxidant machinery in abiotic stress tolerance in crop plants. Plant Physiol. Biochem..

[45] Moore K., Roberts L.J. (1998). Measurement of lipid peroxidation. Free Radic. Res. Commun..

[46] Kleinow T., Himbert S., Krenz B., Jeske H., Koncz C. (2009). NAC domain transcription factor ATAF1 interacts with SNF1-related kinases and silencing of its subfamily causes severe developmental defects in *Arabidopsis*. Plant Sci..

[47] Nakashima K., Tran L.S., Van Nguyen D., Fujita M., Maruyama K., Todaka D., Ito Y., Hayashi N., Shinozaki K., Yamaguchi-Shinozaki K. (2007). Functional analysis of a NAC-type transcription factor OsNAC6 involved in abiotic and biotic stress-responsive gene expression in rice. Plant J..

[48] He L., Bian J., Xu J., Yang K. (2019). Novel Maize NAC Transcriptional Repressor ZmNAC071 Confers Enhanced Sensitivity to ABA and Osmotic Stress by Downregulating Stress-Responsive Genes in Transgenic *Arabidopsis*. J. Agric. Food Chem..

[49] Wu A., Allu A.D., Garapati P., Siddiqui H., Dortay H., Zanor M.I., Asensi-Fabado M.A., Munne-Bosch S., Antonio C., Tohge T. (2012). JUNGBRUNNEN1, a reactive oxygen species-responsive NAC transcription factor, regulates longevity in *Arabidopsis*. Plant Cell.

[50] Yuan X., Wang H., Cai J., Bi Y., Li D., Song F. (2019). Rice NAC transcription factor ONAC066 functions as a positive regulator of drought and oxidative stress response. BMC Plant Biol..

[51] Sheludko Y.V., Sindarovska Y.R., Gerasymenko I.M., Bannikova M.A., Kuchuk N.V. (2007). Comparison of several Nicotiana species as hosts for high-scale Agrobacterium-mediated transient expression. Biotechnol. Bioeng..

[52] Li X., Guo C., Ahmad S., Wang Q., Yu J., Liu C., Guo Y. (2019). Systematic Analysis of MYB Family Genes in Potato and Their Multiple Roles in Development and Stress Responses. Biomolecules.

[53] Zhang X., Henriques R., Lin S.S., Niu Q.W., Chua N.H. (2006). Agrobacterium-mediated transformation of *Arabidopsis thaliana* using the floral dip method. Nat. Protoc..

[54] He K., Zhao X., Chi X., Wang Y., Jia C., Zhang H., Zhou G., Hu R. (2019). A novel Miscanthus NAC transcription factor MlNAC10 enhances drought and salinity tolerance in transgenic *Arabidopsis*. J. Plant Physiol..

