# Supplementary material for: Potato NAC Transcription Factor StNAC053 Enhances Salt and Drought Tolerance in Transgenic Arabidopsis
Source: Int J Mol Sci. 2021 Mar 4;22(5):2568. doi: 10.3390/ijms22052568 (PMC7961516; doi:10.3390/ijms22052568)
Supplement: Supplementary file 1 [file ijms-22-02568-s001.pdf]

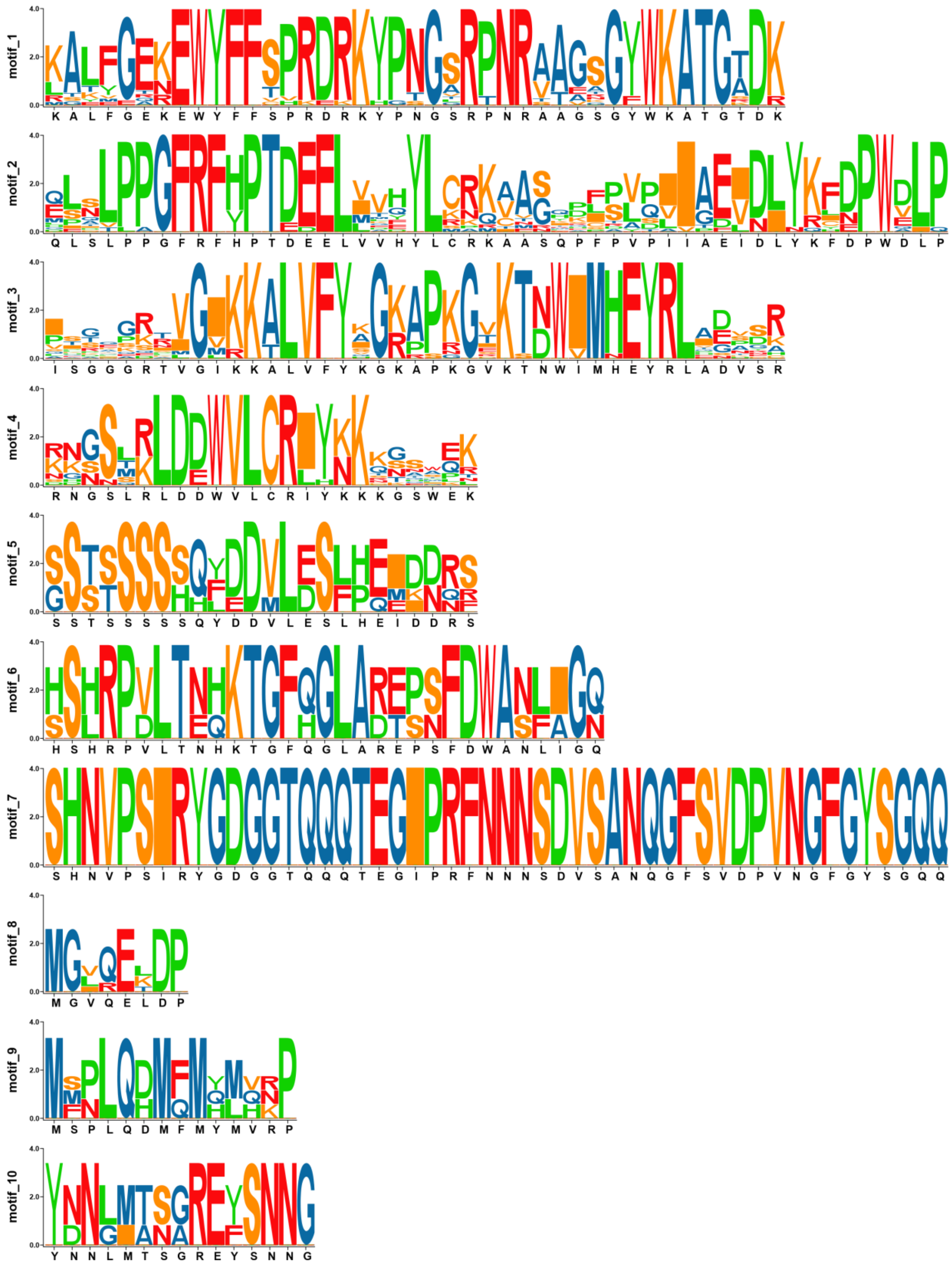

Figure S1: The putative conserved motifs in NAC proteins

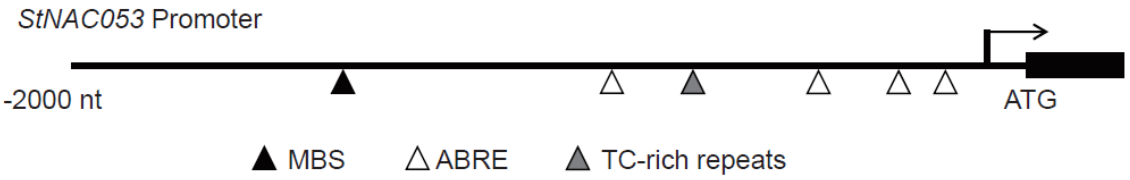

Figure S2 Stress related elements in the promoter regions of *StNAC053* gene

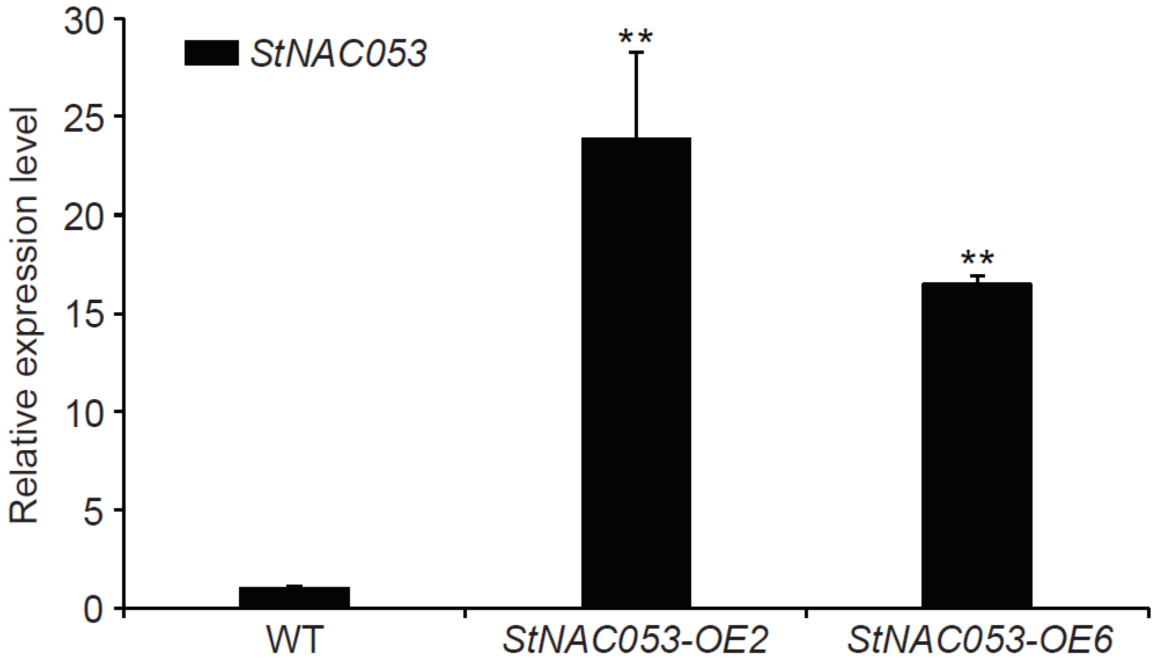

Figure S3 : The expression level of the *StNAC053* gene in wild type and two overexpression lines, the ratios of gene expression levels were calculated relative to the wild type

Table S1 Prediction of NAC binding *cis*-elements in the promoter of stress-responsive genes

Gene	NAC protein binding sites	Number
COR15A	CACG/CGTA/CACGTG	6
DREB1A	CGTA/CGTG/CACG	4
ERD11	CGTG/CACGTG/CACG	3
ERF5	CACG/CGTA/CGTG	7
KAT2	CGTA/CGTG/CACGTG	7
RAB18	CACGTA/CGTA/CGTG/CACG	9

Table S2 The primers used in this study

StNAC053-CDS-F	5'-ATGACAGCAGCTGAATTGCAG-3'
StNAC053-CDS-R	5'-TCAAAAAGGTCTGTGCAGGTG-3'
StNAC053-qRT-F	5'-TTCTACCACCGCTACCTCC-3'
StNAC053-qRT-R	5'-TCGCATGTGAATTCCGGTG-3'
StEF1a_F	5'-ATTGGAAACGGATATGCTCCA-3'
StEF1a_R	5'-TCCTTACCTGAACGCCTGTCA-3'
ACTIN2-F	5'-TGTGCCAATCTACGAGGGTTT-3'
ACTIN2-R	5'-TTTCCCGCTCTGCTGTTGT-3'
DREB1A-qRT-F	5′-AGGAGACGTTGGTGGAGGCT-3′
DREB1A-qRT-R	5′-ACGTCGTCATCATCGCCGTC-3′
ERD11-qRT-F	5′-CCCCTTTGGTAAAGTTCC-3′
ERD11-qRT-R	5′- ATGTCCTTGCCAGTTGAG-3′
ERF5-qRT-F	5′-TTGAAGACGGAACAGAGC-3′
ERF5-qRT-R	5′-AGGAGATAACGGCGACAG-3′
RAB18-qRT-F	5′-GCATAGACTTTGCTCGGGAGT-3′
RAB18-qRT-R	5′-CCGCCAGACGAACCTTCA-3′
KAT2-qRT-F	5′-TGATAATCCTTCCTGCTT-3′
KAT2-qRT-R	5′-CATCATCTATTTCTGCGTTT-3′
Cor15A-qRT-F	5′-CAGTTCGTCGTCGTTTCT-3′
Cor15A-qRT-R	5′-CCAATGTATCTGCGGTTT-3′
